# Maternal body size influences offspring immune configuration in an oviparous snake

**DOI:** 10.1098/rsos.160041

**Published:** 2016-03-16

**Authors:** Gregory P. Brown, Richard Shine

**Affiliations:** School of Life and Environmental Sciences, A08, University of Sydney, Sydney, New South Wales 2006, Australia

**Keywords:** leucogram, maternal effects, immune cells

## Abstract

Like most ectothermic vertebrates, keelback snakes (*Tropidonophis mairii*) do not exhibit parental care. Thus, offspring must possess an immune system capable of dealing with challenges such as pathogens, without assistance from an attendant parent. We know very little about immune system characteristics of neonatal reptiles, including the magnitude of heritability and other maternal influences. To identify sources of variation in circulating white blood cell (WBC) concentrations and differentials, we examined blood smears from 246 hatchling snakes and their field-caught mothers. WBC concentrations were lower in hatchlings than in adults, and hatchlings had more basophils and fewer azurophils than adults. A hatchling keelback's WBC differential was also influenced by its sex and body size. Although hatchling WBC measures exhibited negligible heritability, they were strongly influenced by maternal body size and parasite infection (but not by maternal body condition, relative clutch mass or time in captivity). Larger mothers produced offspring with more azurophils and fewer lymphocytes. The mechanisms and consequences of WBC variation are currently unknown, but if these maternal effects enhance offspring fitness, the impact of maternal body size on reproductive success may be greater than expected simply from allometric increases in the numbers and sizes of progeny.

## 1. Introduction

At birth, animals face an onslaught of parasites, pathogens, antigens and tissue damage. Altricial species are buffered from such challenges by attending parents who supply energy, hygiene or other forms of protection [1,2]. In species that lack parental care, however, offspring must be self-sufficient in defending themselves from pathogens [3]. When the risk of infections or virulence of pathogens is high, we expect strong selection on offspring immune function [4]. Reproducing animals can manipulate the immunocompetence of their offspring via two separate mechanisms. The immune capabilities of a neonate depend not only on its genetic constitution, but also on maternal transfer of other immunomodulatory substances (e.g. antibodies, hormones, antioxidants [5,6]) or behaviours [7], which can alter immune defences of the offspring. In egg-laying species (including in oviparous reptiles), these compounds may be transferred into yolk or albumen and thus incorporated into the developing embryo [6].

A mother's phenotypic traits, including her nutrition and health status, can strongly influence the quantity or quality of immunomodulatory products she transfers, either actively or passively, to her offspring [6]. These products can have both short- and long-term effects on offspring immune function [8]. For example, low energy reserves (as indicated by poor body condition or low litter mass) of a reproducing female may compromise her own immune system [9,10] as well as her ability to allocate nutrients or immunomodulatory substances to her offspring [5,6], which could in turn affect their immune development [8]. Similarly, a female that has produced specific antibodies to pathogens she has encountered may transfer those products to her offspring, providing them with a degree of passive immunity until their own systems are capable of dealing with such challenges [4,11]. Maternal stress during egg provisioning could alter the transfer of immunosuppressive substances, such as corticosterone or testosterone, from mother to offspring [6]. In addition, differing levels of sex steroids allocated to eggs could influence offspring growth rates and indirectly affect their immune investment through energetic trade-offs [6,12,13].

Maternal effects on offspring immune function have been most intensively studied in endothermic vertebrates (birds and mammals), most of which exhibit intensive post-partum parental care [14]. Offspring immune function in these species thus evolves within a context of multifaceted and continuing parental investment into the developing progeny. The situation is very different in ectothermic vertebrates, most of which show no parental care of their offspring [14]. In reptiles, almost nothing is known about the effects of maternal transfer on offspring immune function in reptiles [6]. To place the immune function of neonatal organisms into a broader ecological and evolutionary context, we need to study neonatal immune systems of non-model organisms, ideally in the wild [15–17]. Because interactions among anthropogenic, climatic and disease processes threaten many ectothermic taxa [18,19], information on their ecoimmunology is crucial in assessing risk levels [20–22]. The impact of environmental stressors on maternal health might directly impact offspring quality if a mother's traits directly affect the immune function of her progeny [4,6].

In this study, we investigate sources of variation in constitutive circulating immune defences of hatchling snakes. The indices we use are the concentrations and differential counts (i.e. relative proportions) of white blood cells (WBCs). WBCs are the effector cells of the immune system and play numerous roles in immune surveillance and response [22,23]. The numbers and types of WBCs in circulation provide an indication of immune preparedness and can also predict the magnitude of response to a future immune challenge [23–26]. Although WBC counts may have a genetic basis [27], different immune challenges evoke distinctive changes in the relative proportions of WBC cell types. Thus, differential counts are a mainstay of clinical diagnosis [22,28,29]. The sensitivity of WBC differentials to pathogen exposure and other physiological perturbations (such as stress) makes it difficult to interpret WBC differentials when factors such as prior health, nutrition or pathogen status of an individual are unknown [26,30]. However, if these factors are known, or can be controlled, then it can facilitate interpreting variation in WBC differentials among individuals. For instance, WBC profiles may be an appropriate immune index for comparisons among neonates, whose exposure to pathogens has been limited, compared with older free-ranging conspecifics. Nonetheless, mechanisms underlying WBC differences can be complex, even in neonates [31].

Given the relative paucity of ecoimmunological research on neonatal reptiles, our preliminary goals were to identify patterns of variation rather than to explore their mechanisms or consequences. Specifically, we wished to determine the following:

(i) Are WBC differentials of hatchling snakes similar to those of adults, or do they change in a consistent manner as individuals age [32–34]?
(ii) How strong is familial similarity of hatchling WBC differentials, and are maternal effects primarily genetic [35,36]? Although WBC differentials are labile in response to individual circumstance (see above), heritability and selection studies on production animals have demonstrated significant genetic components [27,37–39]. If baseline WBC profiles have a genetic component, then offspring would be expected to bear a closer resemblance to their sibs and mother than to non-sibs or unrelated adult females.
(iii) Do phenotypic traits of hatchlings (e.g. sex, body size) influence their immune configuration [10,17] as reported in other taxa [40,41]? Do maternal traits (body size, condition, parasite infection, reproductive investment and exposure to stress) influence the WBC configuration of offspring?

## 2. Material and methods

### 2.1. Study site and species

**Figure 1.**
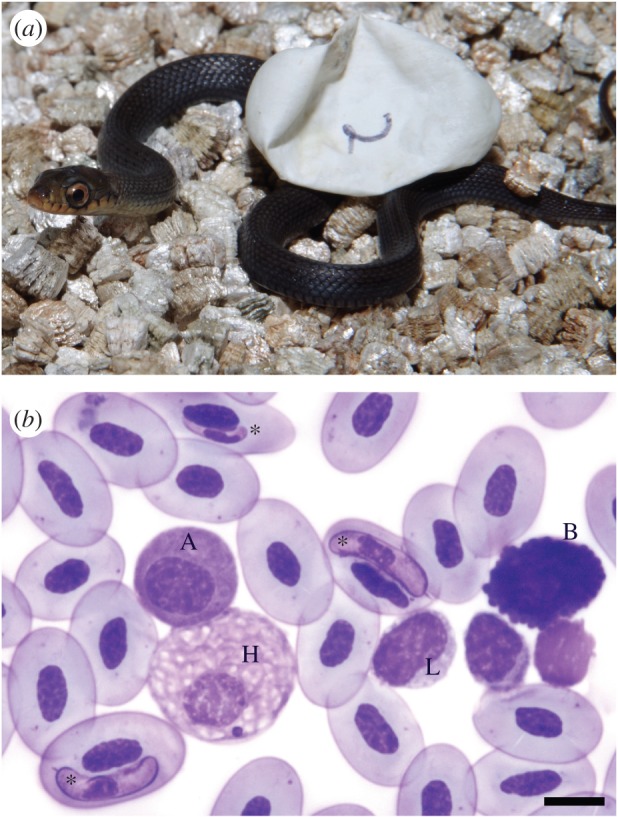
(*a*) Laboratory-incubated hatchling keelback (*Tropidonophis mairii*) and (*b*) keelback blood cells: azurophil (A), basophil (B), heterophil (H) and lymphocyte (L). Asterisks denote haemogregarine parasites infecting red blood cells; scale bar at lower right indicates 10 µm. (Wright stain, 1000×).

Fogg Dam (12.56° S, 131.30° E) is located in the wet–dry tropics of Australia's Northern Territory. Maximum daily temperatures are high (more than 30°C) year round, but rainfall is largely restricted to a six-month (November–April) ‘wet season’ each year. Keelbacks (*Tropidonophis mairii*, figure 1) are non-venomous natricine colubrid snakes in the same phylogenetic lineage as North American water snakes (*Nerodia* spp*.*) and European grass snakes (*Natrix* spp.). Adults are sexually dimorphic in body size (at our study site, females reach 80 cm in snout-to-vent length (SVL) and males reach 68 cm: G.P.B. 2016, unpublished data). The diet of keelbacks consists almost entirely of amphibians [42]. At our study site, keelbacks are active year round. Females begin nesting in April, at the cessation of wet-season rainfall, with peak nesting activity over the following two months [43,44].

As part of a long-term ecological study, we hand-captured gravid female snakes at night during the nesting season. We returned snakes to the laboratory where they were held overnight in individual cloth bags. The next morning, we collected a blood smear by excising 1 mm off their tail tip with a sterile scalpel blade. The droplet of blood that welled from the excision was thinly smeared onto a glass slide, air-dried, fixed in methanol and then stained using modified Wright's stain. Snakes were then measured for SVL and mass, and individually marked by scale-clipping. We held females captive in 35 × 30 × 20 cm plastic cages with a water dish and a nest/shelter box containing damp vermiculite. After each female laid her eggs (1–11 days post-capture; mean = 4.9 days), she was reweighed and then released at her point of capture.

Eggs were collected from nest boxes within 12 h of being laid and individually measured and weighed. Eggs from each clutch were placed together in a sealed plastic bag containing 20 g of vermiculite moistened with 20 ml of water and incubated in an insulated box at 25°C until they hatched (after approx. 60 days). Within 24 h of hatching, each young snake was measured and individually marked as above. In addition, we took blood smears from four to six randomly selected hatchlings from each clutch, in the same way as from adults. All hatchlings were then released at their mother's point of capture. We collected blood smears from a total of 246 hatchling keelbacks, from clutches produced by 49 mothers collected in 2012 (*n* = 1), 2013 (*n* = 6), 2014 (*n* = 20) and 2015 (*n* = 22).

We fitted stained blood smears with coverslips and inspected them under 1000 × magnification in a zigzag pattern to quantify the following.

#### 2.1.1. White blood cell differentials

Each smear was scanned until 100 WBCs were encountered and identified [30] as a basophil, heterophil, monocyte, lymphocyte or azurophil (figure 1). No eosinophils were observed on any slide (these are rare or absent in snakes [28]).

#### 2.1.2. White blood cell concentration

The numbers of WBCs and red blood cells (RBCs) were counted in complete fields of view until at least 1000 RBCs had been enumerated. The WBC count was divided by the RBC count to estimate abundance of WBCs relative to RBCs [34].

#### 2.1.3. Parasitaemia

For blood smears from adult female keelbacks, the RBCs enumerated above were further categorized as being either infected or uninfected with intracellular haemogregarine parasites [45]. To quantify the intensity of haemogregarine infections, we divided the count of infected RBCs by the total RBC count.

### 2.2. Analyses

We ln-transformed count data (WBC differentials, WBC concentration and parasitaemia) to better meet assumptions of regression analysis. We estimated the body condition of snakes using residuals from a regression of ln-transformed mass on ln-transformed SVL. Because of the wide divergence in body size between adults and hatchlings, we performed separate body condition regressions for each group. For adult females, we used post-partum mass to calculate body condition. We measured relative clutch mass (RCM) for each adult female by dividing the total mass of her clutch by her post-partum mass [44].

Differential counts of the five WBC types are not statistically (or biologically) independent, because a proportional increase in one cell type must necessarily result in decreases in other types. To reduce the dimensionality of the five-factor differential count, we used principal component analysis (PCA) on ln-transformed counts of each cell type to provide a single metric that best described the overall pattern of variation in differential counts [46,47]. Although we base our statistical inference on tests using this single omnibus measure of WBC profile, we also present tests on the five individual cell types for post hoc illustrative purposes. Because the tests on individual cell types constitute multiple comparisons, the significance levels of independent variables should be judged accordingly. The specific analyses performed to address our questions were as follows:

(i) To compare WBC traits of the 246 hatchlings to the 49 adult females, we used one-way ANOVAs with age category as the factor.
(ii) To compare levels of variation in WBC parameters within and among families, we first performed one-way ANOVAs with maternal identification (ID) as the factor. To assess familial similarity in WBC differentials in a formal quantitative genetics framework, we also ran an ‘animal model’ [48]. When pedigree information is available on subjects (as is the case for mother–clutch groups), animal models can be used to estimate the genetic components of phenotypic traits [48]. We had maternity information for all the hatchlings in the study, but we did not have paternity information for any of them. One of the hatchlings born in 2014 appeared as a gravid female in 2015, and she thus appears as both an offspring and a parent in the pedigree.
(iii) We performed mixed multiple regressions to assess relationships between physical traits of offspring and mothers on the WBC profiles of the hatchlings. Fixed effects in the model included three hatchling traits (ln-transformed SVL, body condition and sex) and five maternal traits (ln-transformed maternal SVL, post-partum body condition, RCM, ln-transformed parasitaemia and time in captivity prior to oviposition). We included maternal ID as a random effect in the model to accommodate multiple offspring sampled for each female. We nested maternal ID within year to incorporate temporal differences in maternal traits. In initial models, we also included an interaction term between hatchling SVL and sex. In all cases, this interaction was non-significant (all *p* > 0.21) and was thus removed from final models.

We used ASREML software (VSN International Ltd., Hemel Hempstead, UK) to run animal models incorporating random effects (offspring ID and maternal ID) and fixed effects (offspring SVL and/or sex) on WBC concentration and configuration. All other analyses were performed using JMP 11 software (SAS Institute, Cary, NC). We assessed residuals from all analyses to detect violations of assumptions.

## 3. Results

Characteristics of the 49 mother keelbacks, their clutches and the 246 offspring screened for WBC differentials are summarized in table 1. The first principal component (PC1) produced by the PCA explained 41.2% of the variation in ln-transformed WBC differential counts (electronic supplementary table 1). High positive PC1 values describe blood that contains high proportions of basophils and heterophils (loadings of 0.79 and 0.74, respectively) and a low proportion of lymphocytes (loading of −0.90). Loadings on azurophils (0.27) and monocytes (−0.04) were low compared with other cell types. We used PC1 as an omnibus measure of WBC differential in our subsequent analyses.

**Table 1.** Summary statistics of body size, parasitaemia, and clutch and offspring sizes of 49 adult female keelback snakes. SVL, snout-to-vent length; see text for definition of other terms.

variable	*n*	mean (±s.e.)	range
SVL (cm)	49	63.0 (±0.83)	51–78.3
parasitaemia (%)	49	7.0 (±0.74)	0–24
pre-partum mass (g)	49	118.4 (±4.81)	66–202
post-partum mass (g)	49	95.0 (±3.83)	50.2–163
egg mass (g)	49	2.9 (±0.05)	2.13–3.83
clutch mass (g)	49	27.1 (±1.18)	13.8–46
clutch size	49	9.4 (±0.36)	5–15
relative clutch mass	49	0.29 (± 0.008)	0.13–0.39
offspring SVL (cm)	246	15.7 (±0.06)	12.7–18.4
offspring mass (g)	246	2.4 (±0.02)	1.6–3.3

### 3.1. Differences in white blood cell configuration between hatchlings and adults

WBC differentials and concentrations differed significantly between the 49 adult female snakes versus the 246 hatchlings (table 2). Adults had more WBCs relative to RBCs, higher proportions of azurophils, and lower proportions of basophils and heterophils. As a result, adult females had lower PC1 values. The proportions of lymphocytes and monocytes did not differ significantly between adults versus hatchlings.

**Table 2.** Comparison of white blood cell (WBC) parameters between 49 adult female keelbacks versus 246 hatchlings. WBC concentration is the number counted per 1000 red blood cells on blood smears. PC1 is a principal component amalgamating the ln-transformed proportions of the five WBC types. Values are means followed by standard errors. With the exception of PC1, statistical tests were conducted on ln-transformed dependent variables. The last two columns show the result of statistical tests comparing adult female snakes versus hatchlings. Italicized values shows statistically significant effects (*p* < 0.05).

dependent variable	hatchlings	adult females	*F*_1,293_	*p*
WBC concentration	24.6 (±1.09)	31.6 (±2.47)	6.4	*0.012*
WBC PC1	0.19 (±0.09)	−0.98 (±0.20)	29.8	*<0.0001*
% azurophils	13.6 (±0.56)	25.7 (±1.28)	33.2	*<0.0001*
% basophils	19.4 (±0.73)	4.3 (±1.65)	107	*<0.0001*
% heterophils	9.0 (±0.41)	5.4 (±0.92)	21.2	*<0.0001*
% lymphocytes	52.2 (±1.12)	58.4 (±2.53)	3.7	0.056
% monocytes	5.8 (±0.34)	6.2 (±0.77)	0.07	0.788

### 3.2. Familial effects on white blood cell configuration of hatchling keelbacks

**Figure 2.**
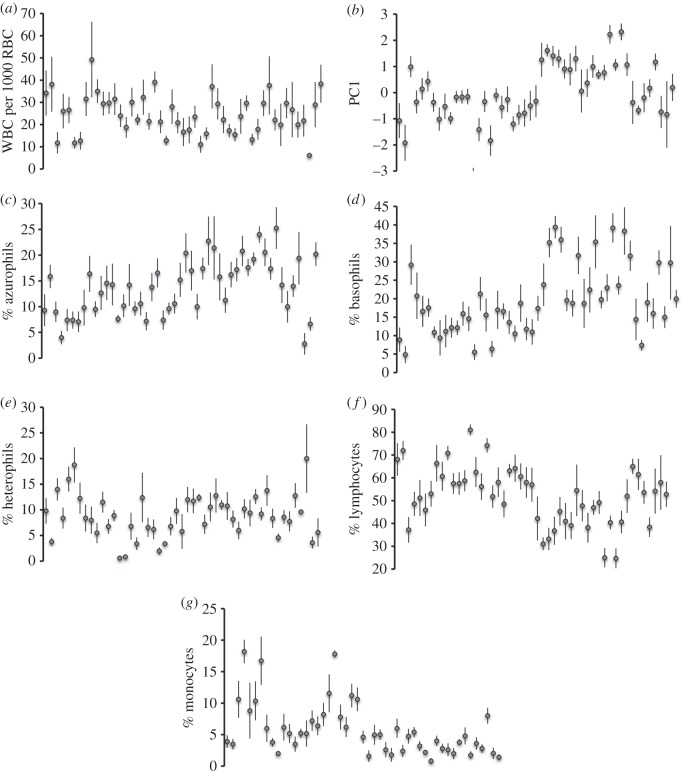
Variation in white blood cell (WBC) measures within and between 49 keelback clutches: (*a*) WBC concentration (no. per 1000 red blood cells), (*b*) principal component (PC1) formed from ln-transformed proportions of five WBC types, (*c*) azurophils, (*d*) basophils, (*e*) heterophils, (*f*) lymphocytes and (*g*) monocytes. The panels show mean values and associated standard errors for each clutch.

**Figure 3.**
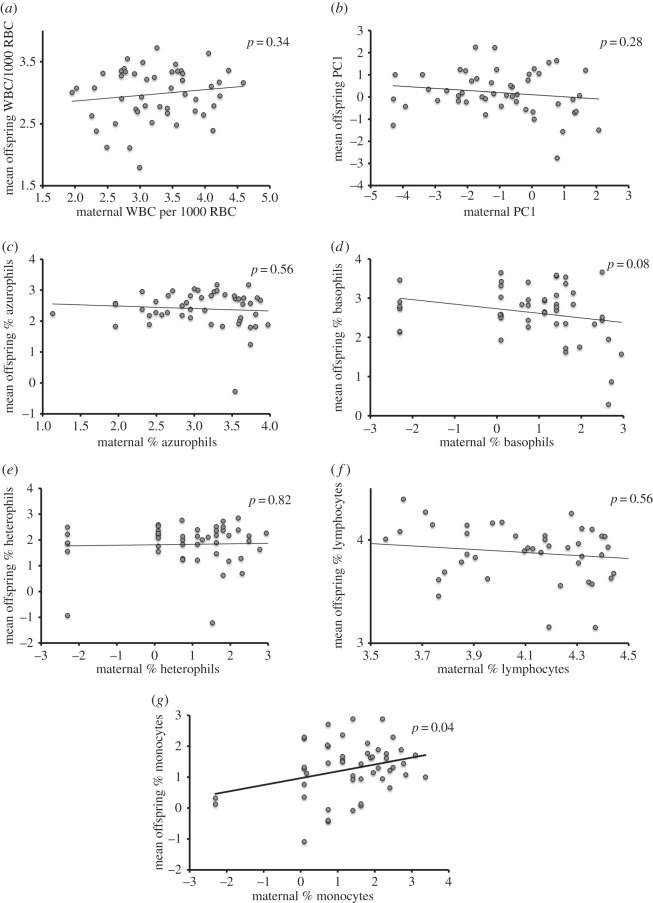
Parent–offspring regression comparing white blood cell (WBC) traits of adult female keelbacks to those of their offspring: (*a*) WBC concentration (no. relative to 1000 red blood cells), (*b*) principal component (PC1) formed from ln-transformed proportions of five WBC types, (*c*) azurophils, (*d*) basophils, (*e*) heterophils, (*f*) lymphocytes and (*g*) monocytes. Values on both axes were ln-transformed then standardized to a mean of 0 and standard deviation of 1. Each data point is based on maternal value and the mean of her offspring values.

Offspring from different clutches varied dramatically in all WBC measures. WBC concentration, PC1 and differential counts of all five cell types varied among the 49 clutches (one-way ANOVA; all *F*_48,197_ > 2.14, all *p* < 0.0001; figure 2). Although WBC characteristics of hatchling snakes were more similar within than among clutches, offspring bore little resemblance to their mothers in these respects (figure 3). Only the proportions of monocytes were similar between mothers and their progeny (parent–offspring regressions: monocytes *F*_1,47_ = 4.47, *p* = 0.04; all other WBC characteristics *F*_1,47_ < 3.18, all *p* > 0.08).

In combination, the above-presented analyses suggest that heritability of WBC traits is low (i.e. little similarity between mother and offspring), but that maternal effects are strong (i.e. less variation within than among clutches). The animal model analyses verified this interpretation. We ran animal models on PC1 and WBC concentration, including offspring traits as fixed effects in both models. For each dependent variable, we used the significant offspring traits identified in (ii) above (sex in the case of WBC concentration, and sex and SVL in the case of PC1; table 3). The heritability estimate derived from the animal model for WBC concentration (conditioned on offspring sex) was low with a large standard error (0.19 ± 0.16, table 3). The estimated maternal effect was of similar magnitude, but with lower variance (0.16 ± 0.07, table 3). The heritability estimate for PC1 (conditioned on offspring sex and SVL) was very low with a large standard error (0.04 ± 0.13, table 3), whereas the maternal effect was moderate with a small standard error (0.36 ± 0.07, table 3). For both WBC traits, including maternal ID as an independent variable significantly improved model fit, indicating the importance of maternal effects in explaining WBC variation [48].

**Table 3.** Animal model analysis on (*a*) white blood cell (WBC) concentration and (*b*) WBC configuration (based on a principal component analysis—PC1—see text) of 246 hatchling keelback snakes. WBC concentration is the number counted per 1000 red blood cells on blood smears. PC1 is a principal component amalgamating the ln-transformed proportions of the five WBC types. Italicized values show statistically significant effects (*p* < 0.05).

trait	variance component	parameter estimate	test statistic	*p*
WBC concentration	*random effects*			
	*V*_maternal_	0.078 ± 0.039	*χ*^2^ = 7.05	*0.008*
	*V*_additive_	0.096 ± 0.082		
	*V*_residual_	0.329 ± 0.071		
	maternal effect	0.155 ± 0.070		
	heritability	0.192 ± 0.16		
	*fixed effects*			
	sex	0.29 ± 0.08	*F*_1,267_ = 14.07	*<0.001*
WBC PC1	*random effects*			
	*V*_maternal_	0.86 ± 0.25	*χ*^2^ = 39.4	*<0.0001*
	*V*_additive_	0.09 ± 0.32		
	*V*_residual_	1.48 ± 0.30		
	maternal effect	0.36 ± 0.07		
	heritability	0.04 ± 0.13		
	*fixed effects*			
	sex	−0.28 ± 0.17	*F*_1,259_ = 2.68	0.10
	SVL	−0.80 ± 0.18	*F*_1,139_ = 20.26	*<0.001*

### 3.3. Effects of offspring and maternal traits on white blood cell configuration of hatchling keelbacks

WBC measures of hatchlings were related to both their sex and body size. Females had higher concentrations of WBCs relative to erythrocytes than did males (means of 27.3 versus 22.7 per 1000 erythrocytes, *p* = 0.0061, table 4). Female hatchlings had higher PC1 values than their male siblings (*p* = 0.0322, table 4), mainly reflecting higher proportions of lymphocytes. PC1 was also affected by hatchling size. Larger hatchlings had lower PC1 values than smaller hatchlings (*p* = 0.0278, table 4), mainly attributable to lower proportions of heterophils and higher proportions of lymphocytes (table 4).

**Table 4.** Multiple regression results on the effects of the phenotypic traits of hatchling keelback snakes and their mothers on the offsprings' WBC profiles. Results are from models incorporating maternal ID nested within year as a random effect. See text for descriptions of dependent variables. With the exception of PC1, tests were conducted on ln-transformed values. Italicized values show statistically significant effects (*p* < 0.05).

dependent variable	source	estimate	d.f.	*F* ratio	prob > *F*
WBC concentration	hatchling sex	0.123	1, 224	7.66	*0.0061*
	SVL	0.001	1, 151	0.00	0.9896
	condition	0.885	1, 103	2.27	0.1346
	maternal SVL	−1. 265	1, 45	2.45	0.1249
	body condition	0.558	1, 41	0.50	0.4850
	RCM	1.428	1, 41	1.13	0.2950
	blood parasites	−0.065	1, 39	0.72	0.4014
	time in captivity	0.005	1, 37	0.04	0.8403
WBC PC1	hatchling sex	−0.167	1, 216	4.65	*0.0322*
	SVL	−0.268	1, 211	4.91	*0.0278*
	condition	0.586	1, 163	0.25	0.6148
	maternal SVL	4.384	1, 50	5.84	*0.0193*
	body condition	−0.305	1, 45	0.03	0.8665
	RCM	−0.773	1, 46	0.06	0.8018
	blood parasites	−0.376	1, 44	4.55	*0.0386*
	time in captivity	−0.066	1, 42	1.14	0.2928
% azurophils	hatchling sex	−0.052	1, 216	1.36	0.2456
	SVL	−0.054	1, 209	0.58	0.4459
	condition	0.320	1, 159	0.23	0.6348
	maternal SVL	2.473	1, 50	5.66	*0.0213*
	body condition	0.218	1, 45	0.04	0.8339
	RCM	−0.846	1, 45	0.23	0.6319
	blood parasites	−0.109	1, 43	1.16	0.2877
	time in captivity	−0.009	1, 41	0.06	0.8002
% basophils	hatchling sex	−0.066	1, 223	0.98	0.3229
	SVL	−0.115	1, 170	1.38	0.2414
	condition	0.198	1, 120	0.05	0.8275
	maternal SVL	1.722	1, 50	1.80	0.1855
	body condition	0.507	1, 45	0.16	0.6895
	RCM	0.192	1, 46	0.01	0.9289
	blood parasites	−0.177	1, 44	2.10	0.1549
	time in captivity	−0.024	1, 41	0.32	0.5746
% heterophils	hatchling sex	−0.095	1, 213	2.63	0.1064
	SVL	−0.181	1, 222	3.73	0.0547
	condition	1.324	1, 181	2.11	0.1479
	maternal SVL	1.258	1, 51	0.71	0.4033
	body condition	−1.523	1, 45	1.04	0.3126
	RCM	−2.919	1, 46	1.33	0.2551
	blood parasites	−0.195	1, 44	1.78	0.1885
	time in captivity	−0.080	1, 42	2.42	0.1269
% lymphocytes	hatchling sex	0.041	1, 216	3.85	0.0511
	SVL	0.072	1, 207	4.94	*0.0273*
	condition	−0.033	1, 156	0.01	0.9161
	maternal SVL	−1.019	1, 50	4.63	*0.0362*
	body condition	0.042	1, 45	0.01	0.9299
	RCM	−0.330	1, 45	0.17	0.6815
	blood parasites	0.086	1, 43	3.52	0.0673
	time in captivity	0.009	1, 42	0.32	0.5736
% monocytes	hatchling sex	0.090	1, 217	1.59	0.2085
	SVL	0.039	1, 205	0.12	0.7264
	condition	1.205	1, 154	1.30	0.2551
	maternal SVL	−3.053	1, 50	3.58	0.0642
	body condition	−0.287	1, 45	0.03	0.8585
	RCM	−0.020	1, 45	0.00	0.9941
	blood parasites	0.231	1, 43	2.18	0.1467
	time in captivity	0.021	1, 41	0.14	0.7073

**Figure 4.**
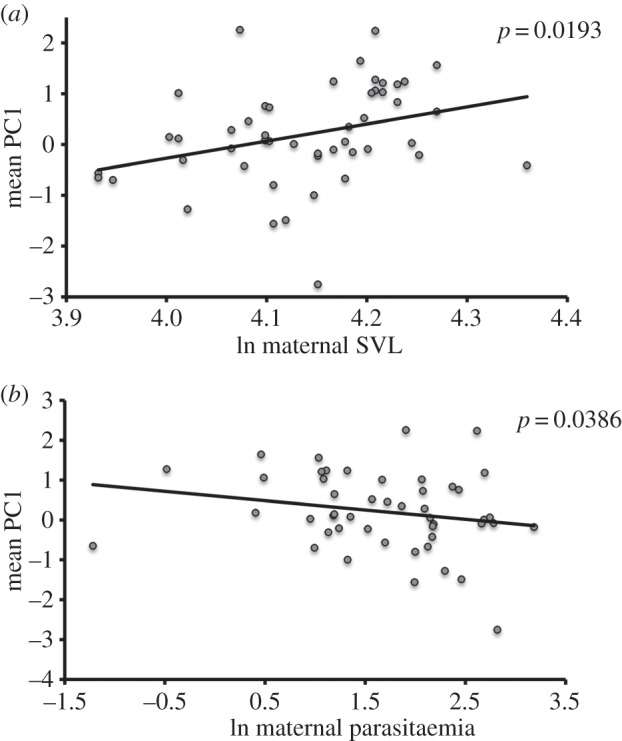
Relationships between white blood cell (WBC) configuration of hatchling keelback snakes and (*a*) maternal body size (ln-transformed snout-to-vent length (SVL)) and (*b*) maternal haemogregarine infection (ln-transformed proportion of red blood cells infected). PC1 is a comprehensive measure incorporating variation in the proportions of five WBC types. Each data point is based on maternal value and the mean of her offspring values.

Hatchling WBC profiles were also influenced by their mother's body size (SVL). Larger mothers produced offspring with higher PC1 values (*p* = 0.0193, table 4 and figure 4), reflecting more azurophils and fewer lymphocytes. A mother's degree of haemogregarine infection also affected the WBC profile of her offspring (*p* = 0.0386, table 4 and figure 4). Females with heavier infections produced offspring with lower PC1 values.

Other maternal traits (body condition, RCM and time in captivity prior to oviposition) had no effect on WBC measures of hatchlings.

## 4. Discussion

We identified several significant sources of variation in the WBC differentials of keelbacks. Our WBC PC variable described an axis corresponding to the relative proportions of granulocytes (basophils and heterophils) versus lymphocytes. This PC is therefore analogous to common WBC differential metrics such as the heterophil : lymphocyte (H : L) ratio and the granulocyte : lymphocyte ratio. These metrics are often used to assess stress levels of individuals [30] or as an indication of innate versus acquired immune configuration [34,49,50]. In reptiles, much of the information regarding variation in WBC counts is anecdotal and based on patterns observed in mammals [28]. Thus, we cannot confidently link WBC configurations to responses to specific immune challenges without experimental assays [46,49] or longitudinal studies that correlate changes in WBC differentials to changes in pathogen levels within individuals. Consequently, we cannot contend that one hatchling WBC configuration confers greater pathogen protection than another. We can only conclude that they are different and plausibly the benefits of different configurations depend on individual circumstances and the pathogen involved. Future work is needed to clarify the mechanisms and consequences of variation in WBC profiles of these snakes.

### 4.1. Differences in white blood cell configuration between hatchlings and adults

The WBC differentials of immunologically naive neonatal keelbacks were dramatically different from those of mature females. Newly hatched snakes had fewer WBCs (relative to RBCs) than adults but much higher levels of basophils. The specific roles of each WBC type are not well understood in reptiles [29]. Basophils release histamine, and their numbers may increase during viral or haemoparasite infection [10,22,26,28]. Given the immunological naiveté of hatchling snakes, the over-representation of basophils among their circulating immune cells plausibly serves a non-specific prophylactic role. As individuals reach maturity, pathogen exposure and sex hormone levels increase, and immune mechanisms may be altered as a result [34,40,49,51,52]. In keelbacks, these ontological changes could potentially alter the importance of basophils relative to heterophils and azurophils. If higher proportions of lymphocytes on blood smears correspond to a more prominent role for acquired immune mechanisms [49], then there is no indication that this role changes with age in keelbacks. Hatchlings and adults have identical levels of circulating lymphocytes but differ dramatically in the proportions of ‘innate’ cell types. However, within lymphoctyes different subtypes (indistinguishable using light microscopy) can be categorized as innate (e.g. natural killer cells) or adaptive (B and T cells), and the relative importance of these subtypes may change with age [53].

### 4.2. Familial effects on white blood cell configuration of hatchling keelbacks

WBC differentials of hatchling keelbacks were similar within clutches but differed among clutches. Although this familial resemblance might suggest that offspring inherit a WBC configuration similar to that of their mother, the WBC profiles of progeny bore little resemblance to that of their mothers. Our animal model analyses verified the negligible heritability of WBC traits but revealed strong maternal effects. Because all eggs in our study were subjected to the same incubation conditions, these maternal effects cannot be due to incubation environment. In natural nests, variation in incubation conditions would inflate among-clutch differences in this respect [54]. Our estimates of heritability are likely to be underestimates, because they assume full-sib relationships within clutches (i.e. single paternity per clutch [55]). However, single paternity is almost certainly unrealistic, given the propensity for snakes in the keelback's lineage to exhibit multiple paternity [56]. If all hatchlings sampled in litters were half-sibs, heritability estimates would be double in value.

WBC differentials vary dramatically over time and in response to numerous external and internal stimuli [29,30]. For instance, stress typically increases the proportion of heterophils and decreases the proportion of lymphocytes [30]. Exposure to pathogens can also differentially alter circulating WBCs [22,28,29]. Given this plasticity in WBC profiles, detecting an underlying genetic component might require more robust pedigree information, including paternity [37].

WBC profiles can also change ontogenetically [32,34], so it would be interesting to compare blood cells of hatchlings and mothers at the same age (i.e. both as hatchlings or both as adults). Although female keelbacks do not transmit their own WBC profile to their progeny, they do seemingly transmit information or substances that modify the WBC differential of their offspring. The immune system is multifaceted, with cellular and humoral components that interact on many levels [22,57]. Thus, maternal transfer of hormones, antibodies or antioxidants to offspring could alter cytokines and other regulatory systems [8] that could potentially affect diverse immune traits, including WBC differentials. If the resulting variance in immune configurations influences offspring survival or reproductive success, it could be a strong target for selection.

### 4.3. Effects of offspring and maternal traits on white blood cell configuration of hatchling keelbacks

At hatching, male and female keelbacks are provisioned with different WBC differentials and concentrations. Compared with males, females have more WBCs relative to RBCs and a higher proportion of lymphocytes among their WBCs (and hence lower PC1). Although male hatchlings are typically larger than females (15.8 versus 15.6 mm SVL in this study), females grow faster and mature at a larger size [58]. Plausibly, the sex difference in WBC differentials of hatchling snakes may be linked to this divergence in growth strategies and life history. In birds and lizards, the level of androgens in eggs has been linked to variation in growth rate and immune function or parasite resistance [13,59]. Sex differences in immune function are widespread, and several hypotheses have been invoked to explain mechanisms and selective forces underlying the divergence [41,52,60,61]. Given high levels of sexual dimorphism in reptiles [62,63], and the immunomodulatory effects of androgens, sex differences in immune function may be widespread even at early ontogenetic stages [13,64]. Sexual divergence in immune configuration could manifest in different risks and costs of pathogen exposure [65–69]. However, available evidence for keelbacks does not indicate sex differences in rates of infection of their two most common parasites, gastric nematodes [70] and haemogregarines [45].

The negative relationship between hatchling body size and PC1 is difficult to interpret. The offspring used for this study were incubated under identical conditions to standardize environmental effects. This was necessary because hatchling phenotypes (SVL, mass) of keelbacks are dramatically influenced by incubation conditions, notably moisture [71]. This plasticity provides an opportunity for future study to decouple the effects of offspring size versus maternal size on WBC differentials [17,72]. Eggs from the same clutch could be incubated under different moisture regimes (to experimentally generate a range of hatchling sizes within each clutch) and their WBC differentials compared. It would also be useful to know whether pathogens in the incubation medium can affect offspring immune configuration. Natural nests can contain bacteria and fungi, and egg albumen contains chemical defences against such organisms [73,74]. The levels of these compounds or the activities of pathogens during incubation may influence traits of the hatchlings [75]. Experimental manipulation of egg hygiene during incubation could clarify the roles of maternal effects versus environmental influences on offspring fitness [76].

Maternal effects were strongly linked to maternal body size and parasitaemia, but not to body condition, reproductive output or time in captivity. The absence of effects of maternal body condition and reproductive output on offspring WBC differentials suggests that the transfer of immunomodulating substances is not linked to maternal energy availability or allocation constraints. Females with heavier parasite infections produced offspring with lower PC1 values (i.e. blood with more lymphocytes and fewer heterophils and basophils). Whether this configuration is more effective at preventing haemogregarine infections in offspring is unknown. Because this parasite appears to have low virulence in keelbacks [45], we might expect that parasites that elicit a stronger immune response in females could similarly modulate WBC differentials or other traits of offspring [11].

The amount of time that female keelbacks were held in captivity prior to laying also did not affect the WBC differentials of their offspring. We collected blood smears only from females at the time of capture, not the time of release, so we cannot directly assess their post-partum stress levels (e.g. H : L ratios [30]). Presumably, the duration of captivity (up to 11 days) was long enough for physiological responses to captivity to manifest. When females were taken into captivity, their eggs may have been at too advanced a stage of development to be altered by maternal stress. Yolk deposition would have been completed, and the shelled eggs already held in the oviduct. Although contact with maternal blood supply provides eggs with oxygen and moisture [77] across the shell, transfer of immunomodulatory compounds might not be possible at such a late stage of development.

The significant effect of maternal body size on WBC differentials of offspring could relate to the mothers' exposure to pathogens other than haemogregarines. Larger females are likely to be older and have ingested more prey items than smaller females. They are thus likely to have been exposed to more environmental and food-borne pathogens and parasites than smaller females [70] and to have had more varied immune experiences.

Because PC1 is partly defined by the ratio of heterophils to lymphocytes (high PC1 scores have high H : L ratios (associated with stress)), can we interpret our results in the context of stress responses [30]? Under this scenario, (i) hatchlings show higher stress levels than adults; (ii) male hatchlings are more stressed than females; (iii) smaller hatchlings are more stressed than large ones; (iv) larger females produced more-stressed hatchlings; and (v) females with more haemogregarines produce less-stressed hatchlings. Some of these patterns (i–iii) seem more intuitively plausible than others (iv,v).

Alternatively, PC1 could be viewed in the context of innate versus adaptive or cheap versus expensive (in relative terms) immune strategies [34,40] (high PC1 values indicate greater reliance on innate/cheaper cells). Under this alternative scenario: (i) hatchlings show greater reliance on innate/cheaper cells than adults; (ii) males show greater reliance on innate/cheaper cells than females; (iii) smaller hatchlings rely more on innate/cheaper cells than larger ones; (iv) larger mothers produce hatchlings that rely more on innate/cheaper cells; and (v) females with heavier haemogregarine infection produce hatchlings that rely more on adaptive/expensive cells. Again, some of these interpretations are more plausible than others. Assessing between alternative mechanisms will require further study.

Regardless of the underlying mechanisms, if the maternal influence on offspring immune configuration has arisen through natural selection, we would expect to observe differential benefits among offspring with different WBC differentials [5,6]. The offspring in this study were individually marked and released into the wild, so future recapture information might identify the benefits associated with different WBC differentials [72]. Recaptures would also provide an opportunity to compare their traits as adults to those of their mother. If the WBC differentials of offspring from larger mothers do provide them with a fitness advantage, then such a link might have important evolutionary and ecological implications. For example, if larger females are able to provide their hatchlings with a fitness-enhancing immune configuration, selection for large female size should increase (over and above any advantage accruing from increased fecundity [78]). Not only would large females produce more and larger offspring [78], but those offspring might be better equipped to fight pathogens. By contrast, large brood size in birds is often negatively correlated with offspring immune function because of increased competition for food among nestlings [79]. Including immunological measures of progeny may thus modify our conclusions about the relationship between maternal body size, reproductive output and evolutionary fitness.

## Supplementary Material

Principal Component Analysis of keelback white blood cell (WBC) profiles.

## Data accessibility

Data have been deposited with Dryad and are available at the following: http://dx.doi.org/10.5061/dryad.9bc55.
