# Supplementary material for: Maternal body size influences offspring immune configuration in an oviparous snake
Source: R Soc Open Sci. 2016 Mar 16;3(3):160041. doi: 10.1098/rsos.160041 (PMC4821281; doi:10.1098/rsos.160041)
Supplement: Principal Component Analysis of keelback white blood cell (WBC) profiles. [file rsos160041supp1.docx]

**Supplementary Table 1.** Results of Principal Component (PC) Analysis of keelback (*Tropidonophis mairii*) white blood cell (WBC) profiles. WBC differential counts were ln transformed prior to analysis. Values under each PC represent loadings (correlations) between the PC and each cell type. PC1 was used in subsequent analyses as the best single overall descriptor of WBC profile.

| **WBC type** | **PC1** | **PC2** | **PC3** | **PC4** | **PC5** |
| --- | --- | --- | --- | --- | --- |
| ln % basophil | 0.79 | 0.11 | -0.33 | -0.44 | 0.24 |
| ln % heterophil | 0.74 | 0.29 | -0.05 | 0.59 | 0.12 |
| ln % monocyte | -0.04 | 0.72 | 0.67 | -0.15 | 0.08 |
| ln % lymphocyte | -0.90 | 0.07 | -0.20 | 0.11 | 0.37 |
| ln % azurophil | 0.27 | -0.77 | 0.54 | 0.02 | 0.19 |
|  |  |  |  |  |  |
| Eigenvalue | 2.06 | 1.22 | 0.89 | 0.57 | 0.26 |
| % variation explained | 41.21 | 24.38 | 17.80 | 11.45 | 5.16 |
